# The impact of a longitudinal mentorship program on medical students: A mixed-methods study

**DOI:** 10.1371/journal.pone.0338476

**Published:** 2025-12-05

**Authors:** Quang Thanh Nguyen, Duc Minh Pham, Hoang Viet Nguyen, Phuong Anh Pham, Vy Huynh Khanh Nguyen, Ngoc Luong Khanh Nguyen

**Affiliations:** 1 College of Health Sciences, VinUniversity, Hanoi, Vietnam; 2 Department of Pediatric Surgery, Vietnam National Hospital of Pediatrics, Hanoi, Vietnam; UTM Skudai: Universiti Teknologi Malaysia, MALAYSIA

## Abstract

**Background:**

Mentorship is a critical element of medical education, shaping specialty preferences, professional identity, and confidence. At VinUniversity in Hanoi, Vietnam, the Longitudinal Mentorship Program (LMP) was designed to provide sustained guidance in a low- and middle-income context.

**Methods:**

This mixed-methods study included 83 medical students from the first two cohorts at VinUniversity. Quantitative data were collected through a cross-sectional survey assessing mentorship’s influence on specialty selection, confidence, and professional identity formation using a 5-point Likert scale (91.2% response rate). Qualitative data were obtained via semi-structured interviews with 14 students, exploring mentorship’s role in career transitions, gender considerations, work-life balance, and role model influences. Statistical analyses, including Spearman correlation and group comparisons, were conducted for quantitative data, while thematic analysis was applied to qualitative findings.

**Results:**

Survey data showed that students rated mentorship highly for enhancing confidence (mean 3.98 ± 1.08), providing useful skills and knowledge (3.98 ± 1.06), and offering valuable insights and advice (4.05 ± 1.09). Mentorship was perceived to moderately influence specialty selection (3.28 ± 1.49). Female students consistently rated their mentors higher, with significant differences for expertise (p = 0.005), professional connections (p = 0.002), emotional support (p = 0.007), and personalized guidance (p = 0.022). Correlation analyses revealed that mentors’ expertise (ρ = 0.488), emotional support (ρ = 0.425), and role modeling (ρ = 0.511) were strongly associated with enhanced confidence and specialty interest. Qualitative findings emphasized the importance of role models, clinical mentors, and personal support, with additional insights into gender considerations, work-life balance, and reinforcement of career aspirations.

**Conclusions:**

This study highlights the critical role of longitudinal mentorship in medical education, demonstrating its impact on career certainty, motivation, and professional identity formation. The findings support the integration of structured mentorship programs into medical curricula to enhance career preparedness and student development. Future research should investigate the long-term effects of mentorship beyond medical school and explore strategies for optimizing mentor-mentee pairing to maximize its benefits.

## Background

Mentorship is a critical component of medical education, guiding medical students through career exploration, professional identity formation, and clinical skill development [1,2]. A well-structured mentorship program can provide students with insights into various specialties, enhance their confidence in clinical decision-making, and offer role models who shape their professional values and aspirations [3–5]. These benefits are particularly salient in early training phases when students often experience uncertainty regarding specialty selection, influenced by factors such as personal interest, gender expectations, lifestyle concerns, and the structure of clinical environments [6,7].

Longitudinal mentorship programs, which provide sustained, individualized guidance throughout the course of medical school, have been increasingly implemented in high-income countries to foster professional growth and reduce the ambiguity surrounding career trajectories [2,8]. Studies from the United States, Canada, United Kingdom and Sweden have demonstrated that such programs can improve clinical readiness, reinforce professional identity, and promote student well-being [1,4,9]. However, there remains limited research evaluating how longitudinal mentorship functions in low- and middle-income countries (LMICs), where structured mentorship opportunities are often underdeveloped or inconsistently applied [10,11].

In the Vietnamese context, recent studies have begun to highlight the nuanced interplay of personal, social, and institutional factors that influence students’ specialty preferences. One study showed that structured interventions, such as career counseling initiatives, positively shaped medical students’ specialty choices by providing guidance and clarifying career trajectories [12]. Another qualitative study emphasized the complexity of students’ decision-making processes, which are shaped not only by intrinsic interest but also by cultural expectations, financial considerations, and gender norms [13]. These findings underscore the need for sustained and contextually relevant mentorship approaches that address both personal and systemic influences in LMIC settings.

In response to this need, VinUniversity, a newly established private medical institution in Vietnam, introduced the Medical Doctor Longitudinal Mentorship Program (LMP) in 2022. In this context, ‘longitudinal’ refers to sustained mentorship provided throughout the duration of the medical school curriculum, spanning six academic years. Grounded in the Nurturing Model of Mentorship [3,5], the program fosters a supportive and open environment where students can seek personal and professional advice, gain exposure to clinical practice and research opportunities, and develop their career trajectories under the guidance of experienced faculty members and clinicians.

The program connects medical students with mentors from affiliated teaching hospitals who are attending physicians or senior lecturers actively involved in undergraduate medical education. Pairing prioritizes alignment of professional interests and availability, and each mentor is assigned no more than two mentees to support individualized guidance. Pairs meet regularly to set goals, review progress, and plan targeted clinical or academic exposure, giving students practical insight into clinical practice, specialty selection, and career planning. International collaborators, including colleagues from the University of Pennsylvania, contributed to the design of the mentorship framework and faculty development resources but did not serve as direct mentors for this cohort. Mentorship sessions also provide a forum to discuss work life balance, professional challenges, and ethical considerations, helping students make well informed career decisions.

Given the growing interest in adapting global mentorship models to LMIC contexts, this study offers an important contribution by examining how medical students perceive the influence of a longitudinal mentorship program on their career decision-making and professional identity formation. To date, few studies in Southeast Asia have systematically evaluated such programs, particularly in private institutions that are experimenting with competency-based and student-centered education.

### Objective of the study

The primary objective of this study is to evaluate the perceived impact of the LMP on the first two cohorts of medical students at VinUniversity. The study focuses on how sustained mentorship influences students’ career decision-making and professional identity formation. In this study, “sustained” refers to an expectation of at least one in-person meeting per month throughout the academic year. Specifically, it examines the ways in which continuous mentor-mentee engagement contributes to specialty selection, confidence development, and long-term motivation throughout medical training.

To achieve this objective, the study addresses the following research questions:

1. How do medical students perceive the influence of longitudinal mentorship on their specialty selection?
2. In what ways does longitudinal mentorship shape students’ confidence and motivation in pursuing a medical career?
3. How does sustained mentor-mentee engagement contribute to the development of students’ professional identity?

By investigating these questions within the context of a structured and evolving mentorship framework, the study aims to elucidate the role of mentorship in supporting medical students’ growth and readiness for future clinical practice.

## Method

### Research design

This study employs an explanatory sequential mixed-methods design, combining quantitative and qualitative methods to assess the impact of the LMP on the first two cohorts of medical students at VinUniversity.

### Ethics approval and consent to participate

This study received ethical approval from both VinUniversity and the Ethics Committee of the Vinmec Healthcare System, under registration number 55/2024/QD-VMEC. Participants were provided with a cover letter that described the study objectives, emphasized the voluntary nature of participation, outlined potential risks and benefits, and included the authors’ contact details. Participants were encouraged to contact the research team with any questions regarding the survey or should they wish to withdraw at any point. Prior to participation, written informed consent was obtained from all individuals. To ensure confidentiality and anonymity, identifying information such as names and email addresses was removed upon submission, and each participant was assigned a unique identification code.

### Program description

Mentors were attending physicians or senior lecturers from affiliated teaching hospitals who met predefined selection criteria: a minimum of three years of post-training clinical practice, ongoing involvement in undergraduate medical education, endorsement by the department chair, and a written commitment to longitudinal engagement for one academic year. Pairing prioritized alignment of professional interests and availability, and each mentor was assigned one or two mentees. Rematching was not planned but could be requested through the program coordinator in exceptional circumstances; no rematches occurred during the study period. Mentors and mentees were expected to meet at least once per month for a minimum of sixty minutes. The first meeting established individualized learning and career goals, documented with program templates. Role expectations included maintaining a regular meeting cadence, responding to mentee queries in a timely manner, facilitating exposure to relevant clinical or academic activities when feasible, providing formative feedback, and referring students to institutional support resources when needed. Meeting logistics, including time and location, were arranged by each pair.

Program preparation emphasized clarity of expectations rather than a stand-alone mentor training course. At the start of the program, mentors received a concise orientation briefing and a written mentor guide that outlined program goals, recommended agendas, goal-setting templates, confidentiality and professionalism standards, and escalation pathways for student well-being concerns. Mentors also had access to existing institutional faculty development workshops that periodically covered mentorship-related skills such as constructive feedback, clinical teaching, and learner support. Mentees attended a brief orientation on preparing for meetings, setting goals, and using the agenda templates. The program did not collect formal meeting logs or reflective documentation, so meeting frequency and content could not be systematically analyzed.

### Quantitative component

A cross-sectional survey was conducted, comprising three sections. The first section collected demographic information, while the second and third sections evaluated the medical students’ experiences with mentorship and its influence on their career decision-making, confidence, and professional identity formation. Participants responded using a 5-point Likert scale, with 1 representing the least influence and 5 the greatest. We used a pragmatic, context specific household income grouping to approximate socioeconomic strata in Vietnam, as no official national standard exists for low, middle, or high income classification; therefore, we referenced national personal income tax brackets, assumed a typical two earner household, and derived household cut points by doubling individual tax thresholds, with all values converted from Vietnamese Dong to USD using the prevailing exchange rate to improve international readability. GPA brackets were taken directly from VinUniversity’s institutional grading system, ensuring internally consistent and transparent categorization without imposing external conversions that may not align with local standards.

To ensure the survey’s validity, items were developed based on an extensive literature review on mentorship and medical career decision-making in diverse contexts, including both high-income and low- and middle-income countries [14–16]. The initial draft of the survey was reviewed by two experts in medical education, who evaluated each item for clarity, relevance, and alignment with the study objectives. Based on their feedback, minor revisions were made to improve wording precision and conceptual consistency. Following expert review, the revised questionnaire was pilot-tested with 10 medical students from cohorts not included in the main study sample. These students were asked to assess the clarity, interpretability, and ease of completion of the items. Their feedback informed further refinements to enhance the tool’s usability and ensure alignment with participants’ educational experiences. While the study did not calculate formal reliability metrics such as Cronbach’s alpha due to its explanatory sequential design and limited sample size, these validation procedures contributed to the overall robustness of the instrument.

### Qualitative component

To complement the survey findings, qualitative data were collected through semi-structured interviews with a subset of students selected via convenience sampling. The design of the qualitative component drew inspiration from the design by Yang et al. [17]. The qualitative study aims to explore how mentorship and role models influence medical students’ career decision-making, specialty selection, and professional identity formation. It examines the impact of assigned mentors and role models, including peers, family members, faculty, and public figures, on medical students’ aspirations and professional growth. Additionally, the study investigates how mentorship experiences during medical school and clerkship shape confidence, career certainty, and specialty preferences. Key factors such as gender, work-life balance, and mentorship dynamics are also analyzed to understand their role in shaping students’ career trajectories.

The thematic analysis of the qualitative component provides deeper insights into the personal and professional influences guiding medical students’ career decisions. It was conducted following the six-phase framework described by Braun and Clarke [18]. Two researchers independently reviewed and coded the interview transcripts using an inductive approach. Initial codes were generated from recurring concepts and patterns emerging directly from the data. Through iterative discussions, codes were organized into preliminary themes, which were then refined, reviewed, and agreed upon by the research team. Discrepancies were resolved through consensus, and thematic saturation was monitored to ensure comprehensiveness. This process helped ensure the reliability and consistency of theme development.

### Study participants & data collection

This study included all medical students from the first two cohorts at the College of Health Sciences, VinUniversity, who participated in the LMP. The sample size was determined based on the total number of eligible students from these cohorts (n = 91). Due to the study’s explanatory sequential nature and the relatively small cohort sizes at VinUniversity, a census sampling approach was employed, inviting all eligible students to participate.

Data collection occurred between August and October 2024, using a specially designed questionnaire developed specifically for this study (see Supplementary Material: S1_Questionnaire), when Cohort 1 was in Year 4 and Cohort 2 was in Year 3 of the MD program. All participants had been engaged in the mentorship program for at least one year at the time of data collection. None of the cohorts had graduated at the time of this study, so the results represent short-term, in-program outcomes rather than post-graduation effects.,. The questionnaire was administered via an electronic survey on Microsoft Forms. Trained student researchers were responsible for disseminating the survey link and monitoring response submissions to ensure completeness and data integrity. The questionnaire was accompanied by a cover letter that outlined the study’s objectives, emphasized the voluntary nature of participation, detailed potential risks and benefits, and provided the authors’ contact information. Written informed consent was obtained from all participants before they voluntarily participated.

For the qualitative component, 14 medical students were selected through convenience sampling, with 7 students from each cohort. Selection was based on participants’ availability and willingness to engage in an interview during the study period. Semi-structured interviews were conducted in person or via secure video conferencing platforms, depending on participant preference and availability. An interview guide was used to ensure consistency across sessions, while allowing for flexibility to explore emerging themes.

Each interview lasted approximately 30–45 minutes and was conducted in English or Vietnamese, depending on participant comfort. Interviews were audio-recorded with prior consent and transcribed verbatim by the first author and two co-authors, who were trained in qualitative interviewing and transcription methods. Transcripts were anonymized to ensure confidentiality. Following transcription, a subset of participants was contacted for member-checking to confirm the accuracy of the transcriptions and preliminary interpretations. This process helped ensure the trustworthiness and credibility of the qualitative data.

Thematic saturation was achieved by the 14^th^ interview, with no new codes or concepts emerging during the final sessions. Saturation was determined through iterative transcript review and regular coding meetings, where the research team confirmed that data redundancy had been reached. This ensured that the qualitative findings reflected the full range of participant perspectives relevant to the research questions.

### Statistical analysis

Quantitative analysis was conducted using R Statistical Software (version 4.4.0), the analysis categorized data where categorical variables were presented as frequencies and percentages, and continuous variables were reported using means and standard deviations. Group comparisons were made using Wilcoxon rank sum and Kruskal-Wallis tests, with statistical significance established at a p-value < 0.05. Additionally, the study assessed the relationship between mentor characteristics and mentee outcomes using Spearman’s rank correlation, with correlation strengths classified as strong (ρ ≥ 0.50), moderate (0.30 ≤ ρ < 0.50), weak (0.10 ≤ ρ < 0.30), or non-significant (ρ < 0.10). No formal correction for multiple comparisons (e.g., Bonferroni) was applied due to the explanatory sequential nature of the study. The primary outcomes analyzed included changes in specialty preference, certainty in career decisions, perceived professional growth, and the impact of role models.

The qualitative component of the analysis was conducted through a thematic exploration of the interview data, utilizing an inductive coding method to reveal underlying themes. This analysis highlighted several significant themes related to career exploration, the influence of mentors, considerations related to gender, aspects of work-life balance, and the reinforcement of specialty preferences. These themes were instrumental in understanding the nuanced impacts of mentorship on the professional development and specialty choices of the participants.

## Result

### I. Quantitative findings

A total of 83 participants took part in the study, 93% for Cohort 1 (43/46) and 89% for Cohort 2 (40/45), the overall response rate was 91.2%. The average age is approximately 22 years. Gender distribution is balanced, with slightly more males (52%) than females (48%). A majority of participants are urban (86%) with varying income levels, the largest group earning above 1,975 USD (42%). Most have a high GPA, with 54% in the 3.20 to 3.59 range. Participant demographic characteristics are summarized in Table 1.

**Table 1:** Participant Demographic Characteristics.

Variable	Overall, N = 83^*1*^	Cohort 1, N = 43^*1*^	Cohort 2, N = 40^*1*^
**Age**	22.05 (± 1.28)	22.47 (± 1.14)	21.60 (± 1.28)
**Gender**			
Female	40 (48.2%)	23 (53.5%)	17 (42.5%)
Male	43 (51.8%)	20 (46.5%)	23 (57.5%)
**Hometown**			
Rural	12 (14%)	8 (19%)	4 (10%)
Urban	71 (86%)	35 (81%)	36 (90%)
**Total Monthly Income**			
< 395 USD	5 (6.0%)	4 (9.3%)	1 (2.5%)
395–789 USD	7 (8.4%)	3 (7.0%)	4 (10%)
790–1,184 USD	17 (20.5%)	8 (18.6%)	9 (22.5%)
1,185–1,975 USD	12 (14.5%)	8 (18.6%)	4 (10.0%)
> 1,975 USD	30 (36.1%)	14 (32.6%)	16 (40.0%)
Undisclosed	12 (14.5%)	6 (13.9%)	6 (15.0%)
**Current Cumulative GPA**			
< 2.00	1 (1.2%)	0 (0.0%)	1 (2.5%)
2.00 to 2.49	1 (1.2%)	0 (0.0%)	1 (2.5%)
2.50 to 3.19	16 (19.3%)	10 (23.2%)	6 (15%)
3.20 to 3.59	41 (49.3%)	23 (53.5%)	18 (45.0%)
3.60 to 4.00	17 (20.5%)	7 (16.3%)	10 (25.0%)
Undisclosed	7 (8.5%)	3 (7.0%)	4 (10.0%)

^1^Values are mean (SD) for continuous variables and n (%) for categorical variables. GPA = Grade Point Average.

**Fig 1:**
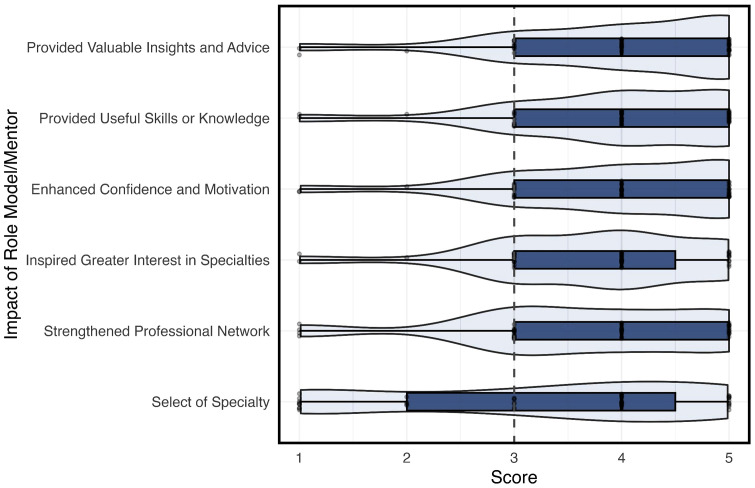
Impact of Longitudinal Mentorship Program on Medical Student.

Fig 1 (Fig1) visualizes the impact of role models or mentors across various dimensions using a 1-5 Likert scale, where 1 represents minimal impact and 5 represents significant impact. The results predominantly show scores in the higher range (3 to 5), suggesting a generally positive impact of mentors in these areas.

Table 2 presents gender-based comparisons in students’ perceived impact of the mentorship program. While mean scores appeared slightly higher among female participants across all measured domains, including the provision of valuable insights, enhancement of confidence, and motivation, these differences were not statistically significant. The greatest perceived benefit, regardless of gender, was in receiving valuable advice and support, whereas the lowest was in influencing specialty selection.

**Table 2:** Gender-Specific Impact of Mentorship Program on Medical Students’ Career Development.

Characteristic	Female, N = 23^*1*^	Male, N = 20^*1*^	Overall, N = 43^*1*^	p-value^*2*^
Selection of Specialties	3.48 (± 1.53)	3.05 (± 1.43)	3.28 (± 1.49)	0.320
Inspired Greater Interest in Specialties	4.00 (± 0.90)	3.50 (± 1.10)	3.77 (± 1.02)	0.151
Provided Valuable Insights and Advice	4.26 (± 0.92)	3.80 (± 1.24)	4.05 (± 1.09)	0.232
Enhanced Confidence and Motivation	4.22 (± 0.80)	3.70 (± 1.30)	3.98 (± 1.08)	0.214
Strengthened Professional Network	3.87 (± 1.06)	3.45 (± 1.32)	3.67 (± 1.19)	0.340
Provided Useful Skills or Knowledge	4.22 (± 0.80)	3.70 (± 1.26)	3.98 (± 1.06)	0.244

^1^Mean (± SD).

^2^Wilcoxon rank sum test.

**Fig 2:**
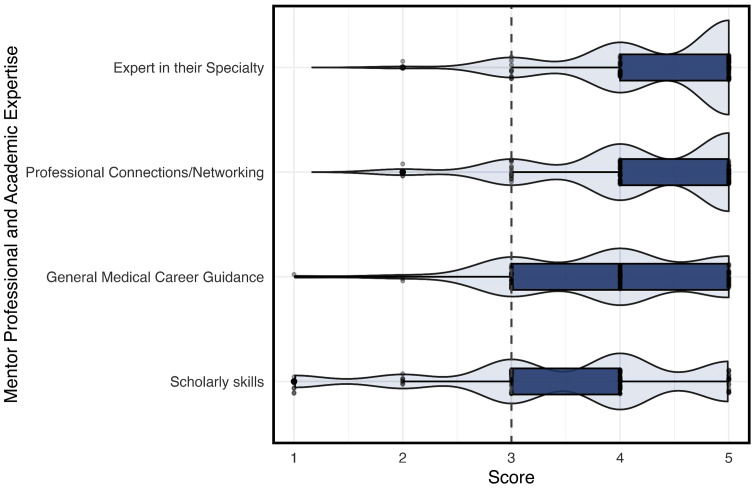
Perceived Professional and Academic Expertise of Mentors by Medical Students.

Fig 2 (Fig2) revealed the evaluation of mentors on professional and academic expertise, including being an expert in their specialty, professional connections/networking, general medical career guidance, and scholarly skills. The scores generally range from 3 to 5, suggesting a positive perception of mentors’ professional and academic capabilities, with expertise in their specialty receiving particularly high ratings.

**Fig 3:**
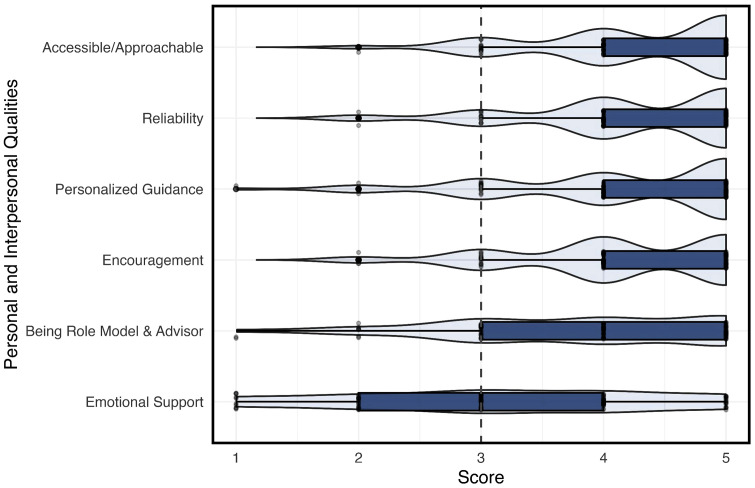
Perceive Personal and Interpersonal Qualities of Mentors by Medical Students.

Fig 3 (Fig3) shows ratings focused on the personal and interpersonal qualities of mentors, including accessibility/approachability, reliability, personalized guidance, encouragement, being a role model and advisor, and providing emotional support. These qualities also receive high ratings, typically between 4 and 5, indicating that mentors are highly valued for their personal and supportive characteristics, with emotional support and being a role model and advisor rated especially high.

Table 3 outlines gender differences in perceived characteristics of mentors among medical students. Women consistently rated their mentors higher than men across various attributes. The most notable differences, with statistically significant results, were in expert knowledge, professional connections, and emotional support, suggesting gender-based variations in mentorship experiences and perceptions.

**Table 3:** Gender Differences in Perceived Mentor Characteristics.

Characteristic	Female, N = 40^*1*^	Male, N = 43^*1*^	Overall, N = 83^*1*^	p-value^*2*^
Expert in their Specialty	4.65 (± 0.62)	4.21 (± 0.80)	4.42 (± 0.75)	**0.005**
Scholarly skills	3.88 (± 1.02)	3.33 (± 1.23)	3.59 (± 1.16)	**0.045**
General Medical Career Guidance	4.18 (± 0.78)	3.70 (± 0.91)	3.93 (± 0.88)	**0.019**
Professional Connections/Networking	4.53 (± 0.72)	3.98 (± 0.89)	4.24 (± 0.85)	**0.002**
Giving Constructive Feedback	4.60 (± 0.63)	4.16 (± 1.04)	4.37 (± 0.89)	**0.049**
Challenges Mentee to Grow	4.00 (± 1.01)	3.86 (± 1.10)	3.93 (± 1.06)	0.588
Communication Skills	4.25 (± 0.84)	4.09 (± 0.95)	4.17 (± 0.89)	0.491
Professional Development Support	4.45 (± 0.99)	4.09 (± 0.87)	4.27 (± 0.94)	**0.011**
Reliability	4.50 (± 0.64)	4.02 (± 0.99)	4.25 (± 0.87)	**0.027**
Encouragement	4.35 (± 0.66)	3.95 (± 1.00)	4.14 (± 0.87)	0.089
Emotional Support	3.60 (± 1.22)	2.84 (± 1.21)	3.20 (± 1.27)	**0.007**
Being Role Model & Advisor	4.13 (± 0.94)	3.56 (± 1.12)	3.83 (± 1.07)	**0.016**
Accessible/Approachable	4.58 (± 0.68)	4.07 (± 0.88)	4.31 (± 0.83)	**0.005**
Personalized Guidance	4.43 (± 0.87)	3.93 (± 1.06)	4.17 (± 1.00)	**0.022**

^1^Mean (± SD).

^2^Wilcoxon rank sum test.

Table 4 presents a Spearman correlation analysis between various mentor characteristics and their impact on providing useful skills and knowledge. Notable positive correlations include expertise in their specialty (0.384), being a role model and advisor (0.358), personalized guidance (0.374).

**Table 4:** Spearman Correlation Analysis of Mentor Characteristics and Their Impact on “Providing Useful Skills and Knowledge”.

Variable	Spearman Correlation^1^	p-value
Expert in their Specialty	0.384*	0.011
Scholarly skills	0.137	0.380
General Medical Career Guidance	−0.026	0.868
Professional Connections/Networking	0.282	0.067
Giving Constructive Feedback	0.247	0.110
Challenges Mentee to Grow	0.030	0.850
Communication Skills	0.242	0.118
Professional Development Support	0.128	0.413
Reliability	0.267	0.084
Encouragement	0.148	0.344
Emotional Support	0.269	0.081
Being Role Model & Advisor	0.358*	0.018
Accessible/Approachable	0.307*	0.045
Personalized Guidance	0.374*	0.013

^1^Spearman Correlation: * p < .05, ** p < .01, *** p < .001.

Table 5 shows a Spearman correlation analysis focusing on how various mentor characteristics impact the enhancement of confidence and motivation among mentees. Key findings include moderate positive correlations for expertise in their specialty (0.488), emotional support (0.425), personalized guidance (0.393), and being a role model and advisor (0.385).

**Table 5:** Spearman Correlation Analysis of Mentor Characteristics and Their Impact on “Enhanced Confidence and Motivation”.

Variable	Spearman Correlation^1^	p-value
Expert in their Specialty	0.488***	0.001
Scholarly skills	0.082	0.602
General Medical Career Guidance	0.139	0.373
Professional Connections/Networking	0.314*	0.041
Giving Constructive Feedback	0.212	0.172
Challenges Mentee to Grow	0.181	0.245
Communication Skills	0.279	0.070
Professional Development Support	0.243	0.116
Reliability	0.386*	0.011
Encouragement	0.120	0.442
Emotional Support	0.425**	0.005
Being Role Model & Advisor	0.385*	0.011
Accessible/Approachable	0.212	0.173
Personalized Guidance	0.393**	0.009

^1^Spearman Correlation: * p < .05, ** p < .01, *** p < .001.

Table 6 presents a Spearman correlation analysis between mentor characteristics and their effect on inspiring greater interest in specialties. Notable strong positive correlations are seen with being a role model and advisor (0.511), moderate correlation in emotional support (0.467), and reliability (0.445), indicating their significant roles in sparking interest in specialties. Expertise in their specialty also shows a substantial correlation (0.430).

**Table 6:** Spearman Correlation Analysis of Mentor Characteristics and Their Impact on “Inspired Greater Interest in Specialties”.

Variable	Spearman Correlation^1^	p-value
Expert in their Specialty	0.430**	0.004
Scholarly skills	0.128	0.413
General Medical Career Guidance	0.250	0.105
Professional Connections/Networking	0.354*	0.020
Giving Constructive Feedback	0.258	0.095
Challenges Mentee to Grow	0.316*	0.039
Communication Skills	0.382*	0.011
Professional Development Support	0.316*	0.039
Reliability	0.445**	0.003
Encouragement	0.298	0.052
Emotional Support	0.467**	0.002
Being Role Model & Advisor	0.511***	<0.001
Accessible/Approachable	0.193	0.216
Personalized Guidance	0.282	0.067

^1^Spearman Correlation: * p < .05, ** p < .01, *** p < .001.

### II. Qualitative findings

#### Theme 1: The impact of role models on medical students’ career aspirations.

Role models, including peers, family members, and public figures, significantly shaped medical students’ career aspirations. They influenced motivation, specialty preferences, and professional values, guiding students in their journey through medical education.

**Subtheme 1.1: Peer and Family Influences: *Peer-Based Motivation*:** Peers emerged as significant motivators for academic effort and career interest, often shaping medical students’ attitudes toward studying and professional aspirations. Many students described peer influence as a catalyst for personal growth.

One male participant noted:

*“My main motivation comes from the people around me… friends and teachers put this pressure on me: ‘I will try to be cool like them.’”* (Student ID: 13, Male)

Some students were introduced to specific medical fields through personal experiences with peers. One female participant recounted how her interest in mental health originated from a middle-school friendship:

*“That friend was very unique but struggled socially. It made me curious about mental health, and I found it fascinating.”* (Student ID: 15, Female)

These findings suggest that peer networks contribute to the early stages of career exploration, exposing students to fields they might not have otherwise considered.

### Family as role models

Family members were frequently cited as key role models, often influencing specialty choices and professional aspirations. For some, relatives’ medical careers shaped their vision of success.

One male student described how his uncle’s international training and leadership role inspired his aspirations:

*“He studied in France, came back, and is now a deputy medical director… That’s the ideal pathway I hope to follow.”* (Student ID: 12, Male). Another male student described how his father’s commitment to continuous education reinforced his own dedication to medicine:

*“He never stops updating himself with modern techniques… He’s a role model of always being curious.”* (Student ID: 17, Male)

Beyond professional success, students admired qualities such as dedication and ethical conduct. A female participant who observed her father’s meticulous patient care shared:

*“He checks on patients after surgery, making sure they are not in pain. He’s very meticulous... and patients truly appreciate that.”* (Student ID: 14, Female)

For some, role models also influenced their perception of lifelong learning and adaptability.

**Subtheme 1.2: Media, famous figures, and specialty experts. *Influence of media and sports icons*:** While some dismissed fictional portrayals as unrealistic, others found inspiration in medical dramas or athletes known for their discipline and perseverance.

One male participant likened Cristiano Ronaldo’s mindset to the determination required in medicine:

*“For me, he has a very strong mentality. Doctors need that too… we have to give everything we have every day in the hospital.”* (Student ID: 17, Male)

### Admiring specialists and entrepreneur-physicians

Certain students were drawn to high-profile specialists who balanced medical expertise with entrepreneurial success.

A male participant described how researching a well-known surgeon shifted his perspective:

*“I wonder how a doctor can be that rich… I researched his career and realized he built his brand with clinics all over the country.”* (Student ID: 16, Male)

Others admired volunteer doctors who provided medical care in resource-limited settings, emphasizing the altruistic dimension of medicine. A female participant mentioned:

*“Doctors in Operation Smile don’t just heal people, they give them a second chance at life. That’s the kind of work I want to do.”* (Student ID: 11, Female)

### Theme 2: The impact of clinical mentors during clerkship

Clinical mentors provided hands-on guidance, helping medical students navigate specialty selection, career transitions, and professional identity formation during clerkship.

#### Subtheme 2.1: Career transition and exploration.

Exposure to clinical practice led some students to reconsider their initial specialty preferences, while others confirmed pre-existing interests.

One male participant, who initially intended to pursue internal medicine, discovered a newfound passion for psychiatry:

*“Initially, I put internal medicine above all else, but now mental health has become equally important… I realized I can cope with patients’ emotional states.”* (Student ID: 23, Female)

Other students used clerkships as a process of elimination:

*“It’s become clearer which specialty I would never choose. I found out which jobs I hate the most and excluded them.”* (Student ID: 22, Female)

Mentors were instrumental in broadening students’ career perspectives by facilitating exposure to different procedures and specialties. One male participant, who switched from pediatrics to internal medicine, credited his mentor for guiding this transition:

*“I wasn’t sure I liked internal medicine, but my mentor encouraged me to try different rotations. That’s how I found out I enjoy procedural work.”* (Student ID: 24, Male)

#### Subtheme 2.2: Gender considerations in specialty selection.

Several medical students expressed concerns about gender-related barriers in specialty selection.

A female student who initially favored cardiology later reconsidered due to perceived gender biases:

*“I liked doing procedures, but cardiology is male-dominant and not very suitable for women in terms of physiology.”* (Student ID: 25, Female)

Conversely, mentorship helped others challenge these gender stereotypes and find empowerment. Mentors who addressed gender concerns in a practical, solution-oriented manner helped students feel more confident in pursuing their preferred specialties.

A female participant described how her mentor influenced her perception of obstetrics:

*“Caring for women’s health is quite important, and I feel that women caring for other women is more empowering.”* (Student ID: 27, Female)

### Subtheme 2.3: Work-life balance considerations

Many medical students reconsidered certain specialties after experiencing their real-world demands.

One female participant ruled out pediatrics after witnessing the intensity of the workload:

*“I eliminated pediatrics because I couldn’t see work-life balance in it.”* (Student ID: 21, Female)

Others realized that internal medicine required a level of responsibility they were not prepared for:

*“I realized making big decisions every day in internal medicine is heavy; I’m not sure I’m ready for that.”* (Student ID: 24, Male)

#### Subtheme 2.4: Reinforcement and self-understanding.

Not all students changed their specialty preferences. For some, clerkship reinforced pre-existing interests:

*“I still like pediatrics and oncology. After clinical rotations, nothing has changed, it’s only been reinforced.”* (Student ID: 27, Female)

Others found greater clarity in their career aspirations.

One female participant, who initially had multiple interests, noted:

*“It didn’t change, but it added to it… the experience helped me understand more about my strengths.”* (Student ID: 15, Female)

## Discussion

This study explored how a structured longitudinal mentorship program influenced medical students’ career decision-making and professional identity formation. The results revealed that mentorship was perceived not only as a source of academic and professional guidance but also as a multidimensional support system that extended to emotional well-being and identity development.

### Mentorship as a bridge between clinical preparation, career exploration, and professional identity formation

Mentorship emerged as a key factor in supporting medical students’ transition from academic learning to clinical application while simultaneously shaping career aspirations and professional identity. Fig 1 showed that students attributed the strongest impact of mentorship to areas such as career guidance, confidence, and motivation. These outcomes underscore mentorship’s instrumental role in bridging the gap between theoretical coursework and real-world clinical experience. Such findings align with Vygotsky’s social development theory, which emphasizes that learners achieve higher competence through support from more experienced individuals [1,4]. In this context, mentors acted as scaffolds who helped students clarify goals, navigate uncertainty, and develop clinical maturity. Structured mentorship programs have similarly been shown to strengthen professional identity and reduce ambiguity in career decision-making [2,5].

Medical students frequently distinguish between mentors and role models. While role models inspired from a distance through exemplary conduct, mentors offered sustained, personalized guidance. This distinction is consistent with existing literature, which positions mentors as practical guides and skill developers, whereas role models often serve as aspirational figures [6,9]. Fig 2 reinforced this differentiation by showing high student ratings for mentors with specialty-specific expertise, clinical competence, and academic experience. Several participants described how their mentors introduced them to specialties they had not previously considered and encouraged academic exploration. These findings mirror prior research highlighting mentorship’s role in expanding students’ scholarly engagement and professional networks [10,11,14–17,19].

Beyond academic and career-related support, mentorship was perceived as essential for emotional and psychological development. As shown in Fig 3, medical students highly valued mentorship qualities such as emotional support, accessibility, and reliability. These interpersonal traits were ranked among the most important mentor characteristics, suggesting that students rely on mentors for guidance not only in career planning but also in navigating the stressors of medical training. Prior studies similarly emphasize that emotionally supportive mentorship enhances student resilience, lowers anxiety, and fosters confidence in clinical environments [11,20].

Additionally, the qualitative findings show that mentorship helps refine specialty interests by either affirming existing aspirations or clarifying paths that are misaligned with personal goals or lifestyle priorities. Students described using mentor guidance to appraise the procedural demands and work–life balance of different fields, an iterative process consistent with literature on how mentorship shapes realistic, individualized career trajectories [21,22].

Students also depicted mentorship as shaping professional identity through three interrelated mechanisms: modeling of professional values, scaffolding of career exploration, and psychosocial support during uncertainty. Role modeling produced concrete shifts in aspirations when mentors demonstrated attainable pathways or challenged stereotypes, for example, “*My mentor encouraged me to try different rotations; that is how I discovered I enjoy procedural work”* [Student 24, male]. Scaffolding occurred as mentors helped students test interests against day-to-day clinical demands, sometimes affirming and sometimes pruning choices: *“It became clearer which jobs I would never choose”* [Student 22, female]. Finally, mentors buffered stressors that strain early identity formation, with approachability and emotional support catalyzing confidence: *“Doctors need a strong mentality. We have to give everything we have every day”* [Student 17, male]. Taken together, these patterns indicate that mentorship influences not only specialty decisions but also the internal work of becoming a physician, linking mentor behaviors to observable gains in confidence, goal clarity, and perceived career fit.

### Gender-specific differences in mentorship perceptions

Gender-related trends were observed in how medical students perceived mentorship. Table 2 indicated slightly higher ratings among female students across several mentorship domains; however, these differences were not statistically significant and should be interpreted with caution. Table 3 did reveal significant gender-based differences in specific mentor characteristics, with female students rating mentors higher in emotional support, personalized guidance, and role modeling. These findings align with prior literature suggesting that women in medicine often value mentorship for both professional affirmation and psychosocial support, particularly in male-dominated fields [23,24].

Some female participants described how same-gender mentorship empowered them to pursue traditionally underrepresented specialties. These experiences align with research that underscores the role of gender-concordant mentorship in fostering confidence and retention among women in procedural specialties [25,26]. Other studies have shown that women are more likely than men to seek mentoring for emotional reassurance and long-term planning [27,28]. However, the findings also highlighted instances where mentorship inadvertently reinforced gender stereotypes. One female student reported being discouraged from pursuing cardiology due to gender-based assumptions. This reflects prior evidence suggesting that the absence of female mentors in some fields continues to contribute to gender disparities in specialty selection [20].

In contrast, male students assigned significantly lower ratings to emotional support, possibly reflecting cultural norms that discourage men from seeking psychosocial guidance [24,29]. These findings underscore the need for mentorship programs to address gendered expectations and ensure that all students receive comprehensive and inclusive support [25,30].

### Mentor-mentee matching and developmental outcomes

The effectiveness of a mentorship program also depends heavily on the compatibility between mentors and mentees. Tables 4–6 provided quantitative insights into how specific mentor characteristics correlated with mentee outcomes. Table 4 showed a positive correlation (0.384) between mentors’ specialty expertise and the development of relevant skills, emphasizing the value of content-specific knowledge. Table 5 demonstrated that emotional support (0.425) and personalized guidance (0.393) were moderately associated with enhanced confidence and motivation. Table 6 revealed the strongest correlation (0.511), showing that mentors who served as role models and advisors significantly inspired students’ interest in particular specialties.

These results suggest that matching should not be random or superficial but based on the alignment of mentor strengths with student needs. However, effective matching involves more than just quantifiable traits. In addition to the positive impacts observed, students described certain practical challenges in engaging with their mentors. These included difficulties scheduling meetings around mentors’ clinical responsibilities and securing appropriate meeting spaces. Occasional differences in expectations also arose, for instance when mentees anticipated research opportunities while mentors prioritized career reflection and professional development. Although these issues were generally resolved informally within each pair, they highlight areas where additional institutional support and clearer guidance could strengthen the effectiveness of the program. Variability in personality, communication styles, and expectations means that even well-matched pairs may not always succeed. To enhance compatibility, institutions should offer flexible pairing options, integrate feedback mechanisms, and allow for reassignment when necessary. Mentors should also receive training in inclusive communication, emotional intelligence, and the unique developmental needs of diverse students.

The mentorship model described here is structured around core elements that are feasible across diverse institutions: explicit mentor selection criteria, brief orientation focusing on inclusive communication and structured goal setting, a minimum meeting cadence with simple goal documentation, and periodic program check-ins. These elements clarify expectations, provide light-touch support, and can be implemented with modest resources. Adaptable components include the intensity of mentor training, the breadth of specialty exposure activities, and the frequency of coordinator oversight, which may be tailored to faculty availability and program scale.

### Recommendations

To enhance the effectiveness of mentorship programs, institutions should adopt a structured set of supports that align with the mechanisms identified in our findings. First, implement brief mentor training that emphasizes inclusive communication, structured goal setting, and formative feedback skills. This preparation strengthens the scaffolding function of mentorship and promotes reliable, growth-oriented interactions. Second, embed faculty development modules that address gendered expectations and work–life balance conversations, since these themes emerged as salient drivers of students’ career decision making. Such preparation is particularly important when gender-concordant matching is not feasible due to mentor availability; in these contexts, mentors can still provide equitable support by using inclusive practices and openly engaging with gender and diversity issues [24,28]. Third, provide simple infrastructure supports to improve reliability and continuity, including scheduling templates, brief goal-tracking forms, and optional peer-mentoring adjuncts that supplement faculty mentorship. These steps are low cost, scalable, and directly target mentor attributes linked to student confidence, specialty exploration, and skill acquisition in our data. Fourth, emphasize interpersonal qualities within mentorship, such as approachability and emotional support, because these qualities substantially influence students’ educational experiences and perceived belonging [20,30]. Fifth, promote exposure to a diverse pool of mentors to mitigate biased guidance and encourage balanced professional development, ensuring that students encounter varied role models and career pathways [19,21]. Finally, expand mentorship models across multiple stages of training so that students receive sustained, longitudinal support throughout the medical education continuum, including transitions into residency and early practice [1,31].

Overall, mentorship should be recognized as a transformative process that shapes not only specialty selection but also professional identity, confidence, and work–life integration. Institutions should embed structured yet adaptable mentorship frameworks across the curriculum to cultivate competent, confident, and resilient physicians [32]. Future research should evaluate long-term outcomes beyond medical school, examine the implementation fidelity of mentor training and infrastructure supports, and explore the experiences of underrepresented minority students to inform more inclusive and equitable mentorship systems [33].

### Limitations of the study

This study has several limitations. First, the small sample size, drawn from the first two cohorts of a newly established institution, limits generalizability. Although the response rate was high (91.2 percent), findings may not reflect broader student populations, particularly in public or larger medical schools. Second, participants in the qualitative arm were selected via convenience sampling, which may have led to overrepresentation of medical students who were more motivated or positively inclined toward mentorship. Third, the reliance on self-reported data introduces the possibility of recall and social desirability biases.

Fourth, while the program was designed as a longitudinal mentorship initiative, the quantitative data were collected cross-sectionally and reflect perceptions at a single time point. Therefore, the study does not assess changes in student outcomes over time or establish causal relationships between mentorship and career development. Relatedly, no baseline measurements were taken to assess students’ initial specialty interests, confidence levels, or professional identity prior to their participation in the mentorship program. This absence limits the ability to determine whether or how mentorship contributed to any changes in these domains.

Fifth, several Likert scale outcomes demonstrated substantial response dispersion, reflected by relatively large standard deviations. In domains where mean scores were close to the neutral midpoint, this variability implies wide margins of error around the estimated mean, such that the uncertainty interval may cross the mid-scale reference value of 3. Consequently, even when statistical tests indicate significance, the magnitude and practical meaning of the effect may be modest and unevenly distributed across students.

Finally, certain program-level aspects were not systematically analyzed in this study. Although mentors were expected to meet at least once a month with mentees and received general faculty development training (which included sessions on constructive feedback and clinical teaching), we did not collect quantitative data on meeting frequency, adherence to structured agendas, or the effectiveness of mentor preparation. Similarly, mentee expectations were not formally measured, even though occasional mismatches with mentor priorities were noted qualitatively. These omissions should be addressed in future work.

Taken together, the findings should be interpreted as representing students’ perceived influence of mentorship, rather than its verified or long-term effects. Future research with pre- and post-program measures, larger sample sizes, and longitudinal designs is needed to more robustly evaluate the developmental impact of structured mentorship programs in medical education.

## Conclusion

The findings of this study suggest that medical students perceived the Longitudinal Mentorship Program (LMP) at VinUniversity to be beneficial in supporting their career decision-making and shaping their professional identity. While based on self-reported experiences, the results highlight mentorship’s perceived role in enhancing confidence, reinforcing specialty interests, and providing emotional and academic support.

These preliminary insights underscore the potential value of structured, ongoing mentorship in medical education, particularly within newer institutions and low-resource settings. Future studies should examine the long-term outcomes of mentorship beyond medical school, explore how mentoring relationships evolve over time, and assess the scalability and adaptability of such programs across different cultural and institutional contexts. Incorporating these findings into educational planning may contribute to the development of more supportive, inclusive, and responsive mentorship frameworks that align with students’ evolving needs throughout their medical training.

## Supporting information

S1 File Study Questionnaire. (PDF)

## Abbreviations

LMP: Longitudinal Mentorship Program
GPA: grade point average
LMICs: low- and middle- income countries
