# Supplementary material for: The impact of a longitudinal mentorship program on medical students: A mixed-methods study
Source: PLoS One. 2025 Dec 5;20(12):e0338476. doi: 10.1371/journal.pone.0338476 (PMC12680140; doi:10.1371/journal.pone.0338476)
Supplement: S1 File — (PDF) [file pone.0338476.s001.pdf]

SURVEY QUESTIONNAIRE

Part 1: Demographic Information

1. Age: __________
2. Gender:
   - o Male
   - o Female
   - o Prefer not to say
   - o Other: __________
3. Cohort:
   - o Cohort 1
   - o Cohort 2
4. Specialty Interest (if any): ________

Part 2: Impact of Mentorship

Instructions: For the following statements, please rate the impact of mentorship on a scale from 1 (No Impact) to 5 (Significant Impact) by circling the appropriate number.

5. Selection of Specialties
   1 2 3 4 5
6. Inspiring Greater Interest in Specialties
   1 2 3 4 5
7. Providing Valuable Insights and Advice
   1 2 3 4 5
8. Enhancing Confidence and Motivation
   1 2 3 4 5
9. Strengthening Professional Network
   1 2 3 4 5
10. Providing Useful Skills or Knowledge
    1 2 3 4 5

Part 3: Characteristics of Mentor

Instructions: Please rate your mentor's performance in the following areas from 1 (Very Poor) to 5 (Excellent) by circling the appropriate number.

11. Expertise in Their Specialty
    1 2 3 4 5
12. Scholarly Skills
    1 2 3 4 5
13. General Medical Career Guidance
    1 2 3 4 5
14. Professional Connections/Networking
    1 2 3 4 5
15. Giving Constructive Feedback
    1 2 3 4 5
16. Challenging You to Grow
    1 2 3 4 5
17. Communication Skills
    1 2 3 4 5
18. Support for Professional Development
    1 2 3 4 5
19. Reliability
    1 2 3 4 5
20. Encouragement
    1 2 3 4 5
21. Emotional Support
    1 2 3 4 5
22. Being a Role Model and Advisor
    1 2 3 4 5
23. Accessibility/Approachability
    1 2 3 4 5
24. Personalized Guidance
    1 2 3 4 5

Qualitative Interview Questions

1. Can you describe the people who have influenced your career choices in medicine?

(This includes role models, mentors, peers, family, or even media figures.)

Probing Questions:

a) Is your assigned mentor also your role model? Can you share an inspiring story about your mentor or role model that had an impact on you?
b) How have family members or peers influenced your decision to pursue medicine or a particular specialty?
c) Have any famous doctors, media portrayals, or public figures shaped your aspirations?
d) What is the difference between a mentor and a role model in your experience?

2. How has your experience with mentorship during medical school influenced your specialty preferences and career decisions?

(This includes formal mentorship, informal guidance, and clinical clerkship experiences.)

Probing Questions:

a) How has your choice of specialty changed throughout your clerkship?
b) Can you describe a moment when mentorship confirmed or altered your specialty interest?
c) Have gender-related factors influenced your specialty choice? If so, did mentorship help address these concerns?
d) Has mentorship helped you assess work-life balance in different specialties?
e) Have you ever ruled out a specialty after learning more about it from a mentor?
